# Successful concomitant open surgical repair of aortic arch pseudoaneurysm and percutaneous myocardial revascularization in a high risk patient: A case report

**DOI:** 10.1016/j.ijscr.2020.07.085

**Published:** 2020-08-15

**Authors:** Roxana Carmen Geana, Ovidiu Stiru, Laura Raducu, Adrian Tulin, Catalina Parasca, Ovidiu Chioncel, Nicolae Bacalbasa, Vlad Anton Iliescu

**Affiliations:** a Emergency Institute for Cardiovascular Diseases Bucharest, Romania; b University of Medicine and Pharmacy, Bucharest, Romania

**Keywords:** Pseudoaneurysm, Aortic arch, Beating heart, Coronary artery disease, Case report

## Abstract

**Introduction:**

We present a case of open surgical repair of an aortic arch pseudoaneurysm (AAP) without the use of hypothermic circulatory arrest in a patient with low ejection fraction and associated coronary artery disease (CAD) and discuss some issues regarding the management of this case.

**Presentation of the case:**

A 69-year-old male with multiple pathologies and history of angina pectoris was transferred to our center from a local hospital with an initial diagnosis of non-ST-segment elevation myocardial infarction. Coronary angiography revealed stenotic lesions affecting all three coronary arteries. Multislice 3D contrast-enhanced computed tomography (CT-scan) revealed a 36 × 27 mm AAP. Endovascular stent-grafting was deemed to be unsuitable due to hostile landing zone. Therefore, the heart team decided for simultaneou treatment of the AAP and percutaneous therapy of CAD. The AAP was excised, and repair was performed with a Dacron patch on beating heart. After the surgical procedure, PCI with drug-eluting stents (DES) was performed on the right coronary artery (RCA) and the left circumflex artery (LCx) in the operating room. The patient's postprocedural course was uneventful and after thirteen days, he was discharged in good shape. 3D CT-scan performed after three month showed no residual AAP.

**Discussion:**

We established this approach as being the most suitable for our case based on favorable circumstances such as: stable CAD, beating heart procedure with low impact on myocardial ischemia, the impossibility of successful placement of an endovascular stent-graft, the presence of a pseudoaneurysm neck with minimum calcification.

**Conclusion:**

We consider the best approach for individual cases is tailoring the treatment plan and the procedure to the patient's specific anatomy and pathology.

## Highlights

• The treatment plan combined open surgical approach for the AAP with PCI for the CAD.
• Both procedures were performed in a hybrid operating room, in the same session.
• The combined procedure was perfomed with a 31.25% calculated risk of mortality.
• Using this approach, we avoided hypothermic circulatory arrest and cardiac arrest.

## 1 Introduction

It has been stated that patients with degenerative aortic arch pseudoaneurysm (AAP) often have associated coronary artery disease (CAD) and also that aortic arch atherosclerosis is commonly found in the population over the age of 55 years [1]. Severe aortic arch atheroma is defined as the presence of an over 4 mm thick, ulcerated or mobile plaque, with prevalence increases with advancing age and is found in patients over 75 years in 20% [2]. Coronary artery disease is also commonly found in the population; in adults, there is a progressive rise with age. Conventional open surgical aortic arch procedures require a sternotomy, cardiopulmonary bypass (CPB) and hypothermic circulatory arrest, thus remaining a surgical challenge with high mortality (2–20%) and morbidity (4–12%) rates depending on the patient's comorbidities, the indication for repair, and the acuity of the presentation [3]. Concomitant AAP and CAD pose an increasing surgical challenge with a high rate of perioperative mortality. Simultaneous myocardial revascularization and aortic arch replacement have a mortality rate of 55%, for which the main causes of death are perioperative myocardial dysfunction, transient or permanent neurological dysfunction, intraoperative bleeding, followed by multiple organ failure syndrome [4,5]. Adequate coronary revascularization is mandatory in order to avoid catastrophic perioperative myocardial infarction and acute cardiac failure. Revascularization of a coronary artery with proximal stenosis, by PCI or CABG, is recommended when a significant myocardial area is subtended [6]. The risk of cerebral embolism during PCI in patients with AAP should not be neglected [4]. In recent years, thoracic endovascular aortic repair (TEVAR) techniques for the aortic arch have evolved significantly and require special consideration. However, an inadequate landing zone for stent-graft deployment due to the very close proximity to the origins of the aortic arch branches makes this type of treatment technically challenging. This case report has been reported in line with the SCARE criteria [7].

## 2 Presentation of case

**Fig. 1:**
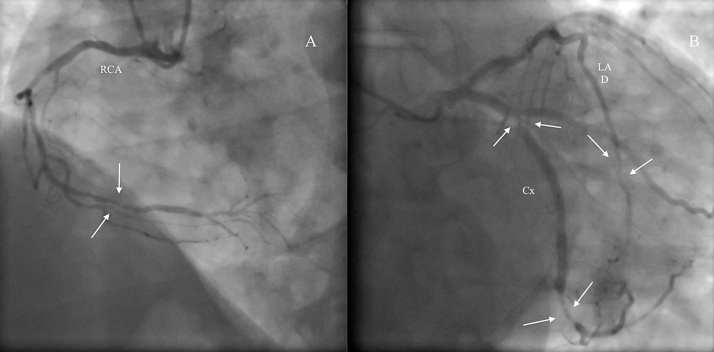
Preoperative coronarography: (A) Coronary angiogram (CAG) shows 70% stenosis in the distal segment of the RCA, (B) Coronary angiogram (CAG) shows 75% stenosis of the the distal segment of the LAD, 80% and 75% stenosis, respectively in the mid and distal segments of the LCx, (CAG - coronary angiogram, LAD - left anterior descending coronary artery, RCA - right coronary artery, LCx - left circumflex coronary artery).

**Fig. 2:**
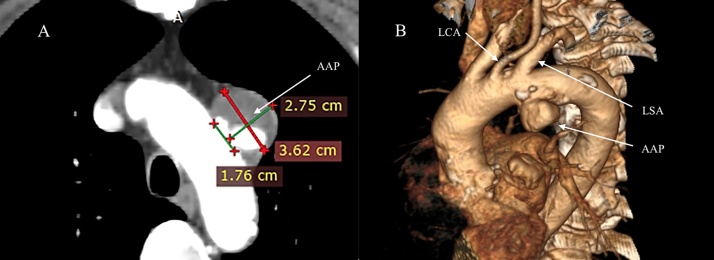
Preoperative contrast enhanced CT scan: (A) CT scan showing the AAP at the level of the aortic arch, (B) 3D CT-scan showing the AAP, with the neck present just caudally from LSA on the lesser aortic arch curvature. (AAP - aortic arch pseudoaneurysm, LSA - left subclavian artery).

**Fig. 3:**
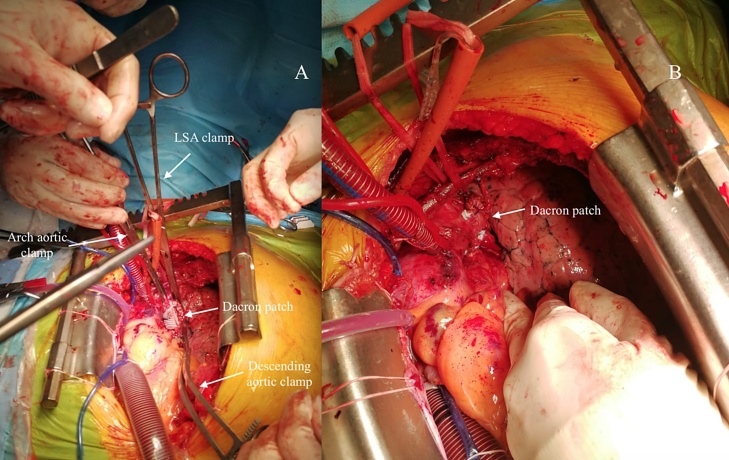
Intraoperator view of aortic arch with Dacron patch repair.

**Fig. 4:**
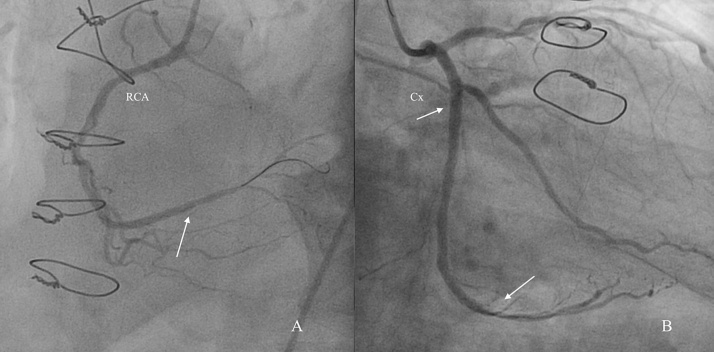
(A) RCA CAG poststenting, (B) LCx CAG poststenting. (CAG - coronary angiogram, RCA - right coronary artery, LCx - left circumflex coronary artery).

**Fig. 5:**
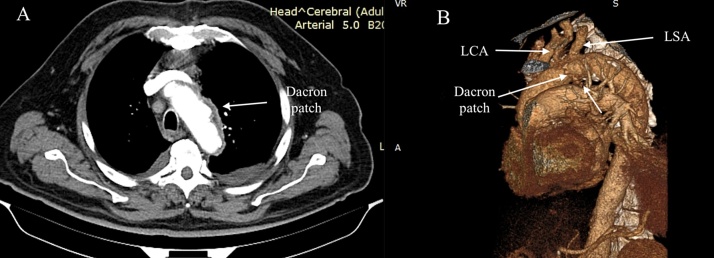
Multislice 3D reconstruction CT scan of the aortic arch after three months showing no evidence of filling or enlargement of the AAP. (AAP- aortic arch pseudoaneurysm).

A 69-year-old male patient previously diagnosed with angina pectoris was referred to the department of cardiac surgery due to the increasing back pain. The patient's medical history included: type II diabetes mellitus, therapeutically neglected arterial hypertension, chronic obstructive pulmonary disease, obesity, chronic coronary artery disease, as well as carotid artery atherosclerotic disease with 70% stenosis in the left carotid artery (LCA). He had no history of stroke, transient ischemic attacks, syncope or thoracic trauma before the presentation. Coronary angiography (CAG) showed left dominant coronary system with triple vessel coronary disease: 75% stenosis of the distal segment of left anterior descending coronary artery (LAD), left circumflex coronary artery (LCx) in the mid and distal segments had stenosis of 80% and 70%, respectively, and 70% stenosis of distal segment of the right coronary artery (RCA) (Fig. 1). Transthoracic echocardiography (TTE) performed at admission showed: severe decrease in left ventricle ejection fraction (EF = 30%), akinesia of the basal segment of the inferior wall and in 1/3 posterior wall, dilated (47 mm) right ventricle with global systolic dysfunction, mild mitral regurgitation, mild tricuspid regurgitation, pulmonary artery systolic pressure (sPAP) of 45 mmHg. Doppler echographic assessment of femoral and carotid arteries confirmed the presence of non-stenotic echo-lucent plaques in both femoral arteries and 70–75% stenosis on the LCA. The ECG showed signs of atrial fibrillation and left ventricular hypertrophy. Three-dimensional analysis of the preoperative computed tomography (3D CT-scan) was used to establish the dimension of the pseudoaneurysm, the distances between the pseudoaneurysm and the aortic arch branches, as well as to identify any signs of intramural hematoma at the level of the proximal seal zone. Apart from the complex lesions of the coronary arteries, 3D CT-scan revealed an AAP, with the neck present just caudally from the left subclavian artery (LSA) on the lesser aortic arch curvature which had a maximum diameter of 36 × 27 mm (Fig. 2). Diameters of the ascending aorta, aortic arch, and descending aorta were 38 mm, 36 mm, and 28 mm, respectively. The distance between the pseudoaneurysm and the LCA origin from the aortic arch was 10 mm. CT scan images were sent and processed by an outside service for preoperative planning and proper sizing of stent-graft. The endovascular aortic arch repair was not recommended due to the lack of adequate landing areas for stent-graph placement. The patient's condition was critical due to the high probability of death due to rupture of the pseudoaneurysm of the aortic arch. Calculated Euroscor II revealed 31.25% risk of in hospital mortality. The multidisciplinary meeting, including cardiac surgeons, interventional cardiologists, and anesthesiologists, discussed the possible treatment strategies for the patient considering the 3D CT-scan, coronarography, Doppler ultrasound echography, and echocardiography image. We offered the patient open surgical repair of the aortic arch pseudoaneurysm and PCI for the lesions of the RCA and LCx, as the lesion on the LAD was considered unsuitable for PCI. The considerable risks of surgery regarding perioperative mortality and its possible complications, as well as the expected natural history without surgery, were communicated to the patient. The patient agreed to undergo the treatment established by the multidisciplinary team. The combined procedure was performed in a hybrid operating room by a multidisciplinary team of cardiac surgeons and interventional cardiologists. Intra-operative standard monitoring of the patient included: invasive blood pressure monitoring via the left radial artery, continuous electrocardiography (ECG), pulse oximetry, diuresis, capnography, and central venous pressure monitoring via a triple lumen central venous catheter. Intraoperative specific monitoring for aortic arch procedures included: two body temperature measurement sensors, rectal and esophageal, an additional arterial blood pressure monitoring via the left femoral artery and near-infrared spectroscopy (NIRS) for continuous monitoring of the cerebral oxygenation. A median sternotomy was performed, and CBP was initiated using a bifurcated arterial line with double arterial cannulation of the ascending aorta and the left femoral artery and right atrial cannulation with a dual-stage venous cannula. The left side of the heart was vented through the right superior pulmonary vein. Although the pseudoaneurysm was adherent to the vessels of the arch, with meticulous dissection of the surrounding tissues, we were able to mobilize all the branches of the aortic arch and their proximal segment before initiating the repair of the pseudoaneurysm. The aorta was first cross-clamped immediately after the origin of the LCA and a second clamp at the level of the aortic isthmus allowing us to perform the repair of the pseudoaneurysm on beating heart (Fig. 3). The pseudoaneurysm was opened and removed until the neck. The aortic rim of the neck was only slightly calcified and was considered to be healthy, and thus we considered safe to perform the repair with a 3/4 cm oval Dacron patch. The time of CPB was 54 min, and the nasopharyngeal temperature was maintained at 35 °C during the entire procedure. After weaning of CPB the PCI with DES was performed for the RCA, and LCx lesions (Fig. 4). The lesion on the LAD was considered unsuitable for PCI due to the extremely calcified aspect of the lesion, which had a very high risk of rupture during the procedure. The patient's postoperative course was unremarkable. He was extubated at 12 h after the procedure, without signs of myocardial ischemia and transient or permanent neurologic injury. In the first postprocedural day chest drainage was of 450 mL with significant haemoglobin level decrease for which the patient received two red cell units and four units of frozen fresh plasma, without the need of surgical revision, a grade II complication according to the Clavien-Dindo classification. Considering this complication, antiplatelet therapy with Clopidogrel was initiated on the second postprocedural day and maintained for 12 months postprocedural. Chest drains were removed on the third postprocedural day and the patient was discharged from the hospital on the 14th postoperative day. At three months after this procedure, a follow-up CT scan with 3D reconstruction was performed and revealed an excellent result, without residual arch pseudoaneurysm (Fig. 5).

## 3 Discussion

Pseudoaneurysms of the thoracic aorta result from transmural disruption of the aortic wall and can be secondary to trauma, vasculitis, infection, previous cardiac surgery or atherosclerosis [8]. The options for suitable treatment of coronary artery disease and aortic arch repair (one-stage or two-stage) are still under debate. Conventional open aortic arch patch repair remains an option for aortic arch pseudoaneurysms when endovascular repair is not a valid option [9,10]. In recent years, conventional open surgical repair has been replaced by hybrid procedures [11]. Open arch replacement with frozen elephant trunk or open arch replacement with retrograde thoracic endovascular aortic repair (TEVAR) have proven to be effective [12,13]. The main concept of hybrid procedures which requires a remaining sufficient landing zone after endograft deployment in order to obtain an adequate sealing zone was not achievable [14]. Open aortic arch repair requiring CPB and hypothermic circulatory arrest remains a valid option. Optimal treatment for patients with concomitant AAP and CAD is dictated by the presence of either critical coronary arteries disease or by impending ruptured aortic pseudoaneurysm. Simultaneous aortic arch repair and surgical myocardial revascularization has been proposed, but the results of this approach were disappointing. Another approach includes two-stage surgery with myocardial revascularization before arch repair or arch repair before myocardial revascularization [15]. If myocardial revascularization had been performed first, the risk of rupture of the pseudoaneurysm in the postoperative course would have been significantly increased. In the second approach, if the arch repair had been performed first, the intraoperative changes of the patient's hemodynamic status could have aggravated the existing myocardial ischemia with a worse effect on the cardiac function. The most favorable situation seems to be represented by a combined treatment, PCI for the coronary lesions and endovascular treatment for the aortic arch pseudoaneurysm. The patient's coronary lesions were suitable for either CABG or PCI as suggested by the SYNTAX I and II scores. Considering the high risks of a combined surgical procedure, in our patient the heart-team opted for PCI as treatment for the coronary arteries lesions. In our case, the surgical repair of the aortic arch pseudoaneurysm was successfully performed on beating heart by closing the lesion with a Dacron patch. By using this technique, we were able to avoid hypothermic circulatory arrest, cardiac arrest, myocardial protection, as well as major reconstructive aortic arch surgical procedures. Using secvential aortic arch repair followed by PCI, we were able to offer protection against the uncontrolled expansion and possible rupture of the pseudoaneurysm and also to avoid the risk of distal embolization emerging from the pseudoaneurysm's content. We established this approach as being the most suitable for our case based on favorable circumstances, such as: stable CAD, beating heart procedure with low impact on myocardial ischemia, the impossibility of successful placement of an endovascular stent-graft, the presence of a pseudoaneurysm neck with minimum calcification.

## 4 Conclusion

In conclusion, this case highlights the importance of individualised approach for complex cases with the treatment plan devised according to the patient's specific anatomy and pathology.

## Declaration of Competing Interest

All authors report no conflicts of interest.

## Funding

We have no sources of funding to declare related to this case report.

## Ethical approval

"Prof. dr. C. C. Iliescu" Emergency Institute for Cardiovascular Diseases, Cardiac Surgery Department, conducted a review of the submission and concluded that activities described in this study do not constitute human subjects research as the project does not involve identifiable private information from the patient and the subject has consented to the publication of their case. A letter by Medical Director of the "Prof. dr. C. C. Iliescu" Emergency Institute for Cardiovascular Diseases Bucharest, Romania was provided and is available upon request.

## Consent

Written consent was obtained from the patient for publication of this case report and accompanying images. A copy of the written consent is available for review by the Editor-in-Chief of this journal on request.

## Author contribution

Ovidiu Stiru: managed the patient and performed the surgery, wrote the manuscript, revision, corresponding author.

Roxana Carmen Geana: managed the patient and performed the surgery, revising critically, wrote the manuscript.

Adrian Tulin: review and editing, wrote the manuscript.

Laura Raducu: patient care, revision.

Catalina Parasca: patient care, revison.

Nicolae Bacalbasa: wrote the manuscript, revison.

Ovidiu Chioncel: revising critically.

Vlad Anton Iliescu: the supervisor, revising critically.

All authors read and approved the final manuscript.

## Registration of research studies

N/A.

## Guarantor

The Medical Director of the "Prof. dr. C. C. Iliescu" Emergency Institute for Cardiovascular Diseases Bucharest, Romania.

Ovidiu Stiru MD PhD.

## Provenance and peer review

Not commissioned, externally peer-reviewed.
